# Joubert Plus syndrome in a child with Dandy-Walker malformation and occipital cephalocele: A case report

**DOI:** 10.1016/j.radcr.2025.05.103

**Published:** 2025-06-27

**Authors:** Tsega Hagos Mehari, Bemnet Mengesha Tessema, Redaie Girmay Gebrekidan

**Affiliations:** Department of Radiology, Mekelle University, Mekelle, Ethiopia

**Keywords:** Molar tooth sign, Joubert Plus syndrome, Dandy-Walker malformation, Occipital cephalocele, Magnetic resonance imaging, Radiology

## Abstract

Joubert syndrome is a rare congenital cerebral developmental malformation that primarily affects the cerebellum. It is characterized by the absence or underdevelopment of the cerebellar vermis and a malformed brain stem (molar tooth sign) on a transverse view of the head magnetic resonance imaging scan. Major clinical features of Joubert syndrome are typically present in early infancy, with most children showing delays in gross motor milestones. When associated with other posterior fossa congenital malformations, it is categorized as Joubert-Plus syndrome. The co-occurrence of both disease entities in one patient has been reported only in a few case reports. In addition, the cystic dilatation of the posterior fossa in Dandy-Walker malformation can mask recognition of the molar tooth sign unless there is a high index of suspicion. We hereby present our experience with diagnosing a combined Joubert syndrome and Dandy-Walker malformation in a 3-year-old girl using magnetic resonance imaging and CT scan after she presented with generalized floppiness since birth.

## Introduction

Joubert-Plus syndrome, first introduced by Quisling et al. in 1999, refers to cases of Joubert syndrome accompanied by additional anomalies involving the mesencephalon or the caudal fourth ventricle, such as Dandy-Walker malformation [1]. Although not formally standardized, this terminology has been employed in several case reports to emphasize the overlapping neurodevelopmental anomalies [2,3]. In contrast, Joubert Syndrome Related Disorders (JSRD) encompass a broader spectrum of conditions in which the classic features of Joubert syndrome are accompanied by abnormalities in other organ systems beyond the central nervous system [4].

The coexistence of Joubert syndrome and Dandy-Walker malformation is uncommon. The overlapping radiologic and clinical features may obscure the identification of each condition individually, complicating accurate classification within the spectrum of posterior fossa malformations.

## Case presentation

A 3-year-old girl was referred to our hospital for neuroimaging after she presented with delayed developmental milestones, generalized hypotonia, and an occipital swelling since birth. The child was born at 39 weeks of gestation via spontaneous vaginal delivery with no significant perinatal complications, except for presence of occipital swelling. Her mother had a routine, uneventful antenatal follow-up at a nearby health center and supplemented with folate during the first two months, but an obstetric ultrasound examination was not done during the pregnancy due to setup limitations. The patient is the firstborn to her parents. Family history is unremarkable with no history of known consanguinity or neurologic disorder.

On physical examination, the patient was noted to have poor head control and a generalized low muscle tone. She was unable to walk without support. A soft swelling was observed in the occipital region, with no evidence of fluid discharge. Her vital signs, including respiratory rate, were normal. There were no dysmorphic facial features. Ophthalmologic evaluation was also unremarkable. Based on these findings, a central cause of hypotonia was suspected.

Initial laboratory investigations of the patient, including complete blood count, renal and liver function tests, were all within the normal range.

An abdominal ultrasound was done to check for possible multisystem involvement. It showed no abnormalities in the kidneys, liver, or other abdominal organs.

**Fig. 1:**
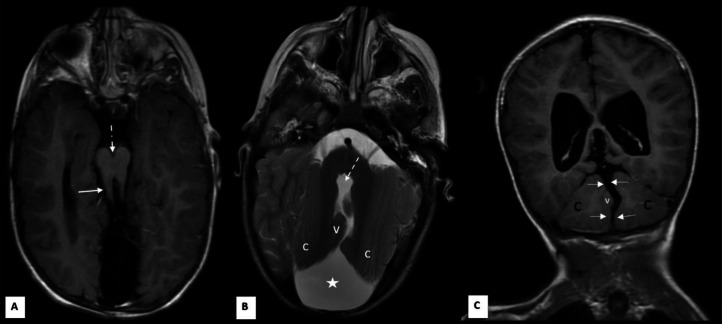
Joubert Plus syndrome. Axial T1-weighted (A), axial T2-weighted (B), and coronal T1-weighted (C) MRI images. Panel (A) demonstrates the elongated superior cerebellar peduncles (solid arrow) and deep interpeduncular fossa (dashed arrow), characteristic of the molar tooth sign. Panels (B and C) show the fourth ventricle in direct communication with a large CSF-filled posterior fossa cyst (five-point star), resulting in thinning of the overlying occipital calvarium. In panel (C) the cerebellar hemispheres (highlighted with the letter “c”) are separated by a deep midline cleft and exhibit an interdigitating appearance (solid arrows). Notice the vermis appears markedly hypogenetic with elevated fastigium, denoted by the letter “V”.

**Fig. 2:**
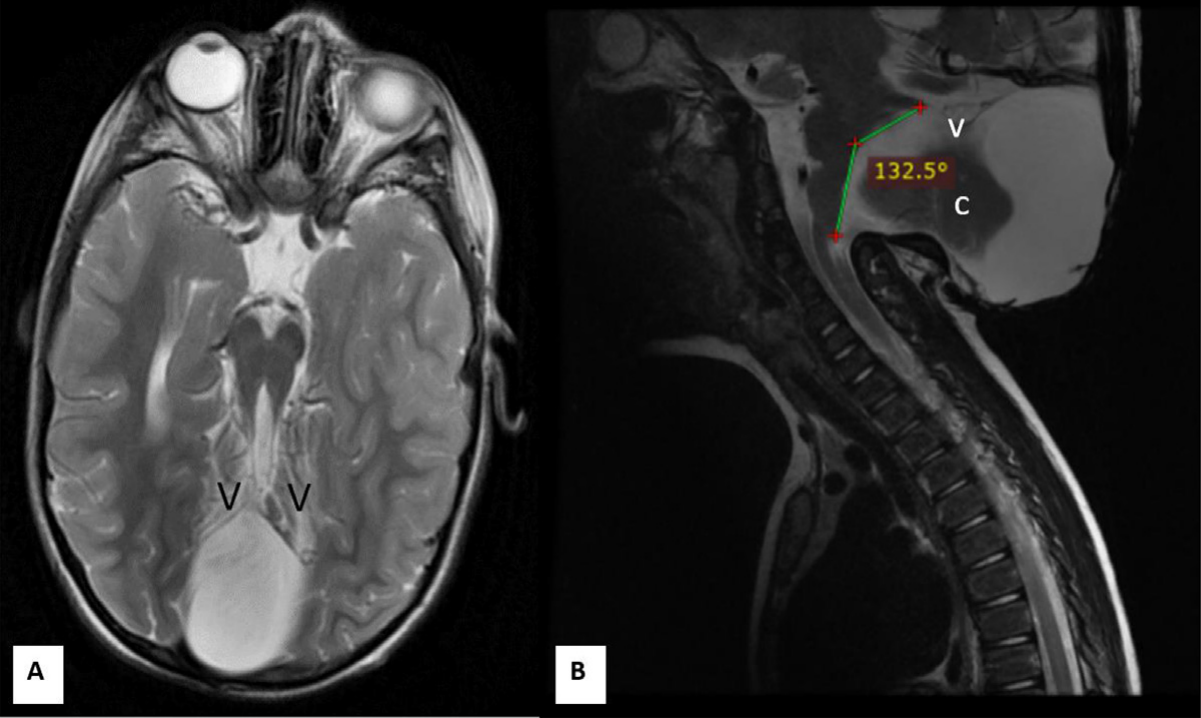
Joubert Plus Syndrome. Axial (A) and sagittal (B) T2-weighted MRI images. The tegmento-vermian angle is markedly elevated, measuring approximately 132.5°, consistent with vermian hypogenesis. The caudal and posterior portions of the vermis are absent, with only a rudimentary superior remnant visible (indicated by “v”). Due to the interdigitating midline configuration, some portion of the cerebellar hemisphere appears in the midline (labeled “c”).

MRI of the brain showed a nearly absent, markedly hypogenetic cerebellar vermis with thickened and horizontally oriented superior cerebellar peduncles, forming the classic “molar tooth sign” (Fig. 1A-C, Fig. 2A and B). In addition, the tegmentovermian angle was increased measuring 132.5 degrees (Fig. 2B).

The supratentorial brain structures, including the corpus callosum, were unremarkable. No signs of ventricular prominence, transependymal CSF flow, or sulcal effacement were seen on T2-weighted and FLAIR sequences.

**Fig. 3:**
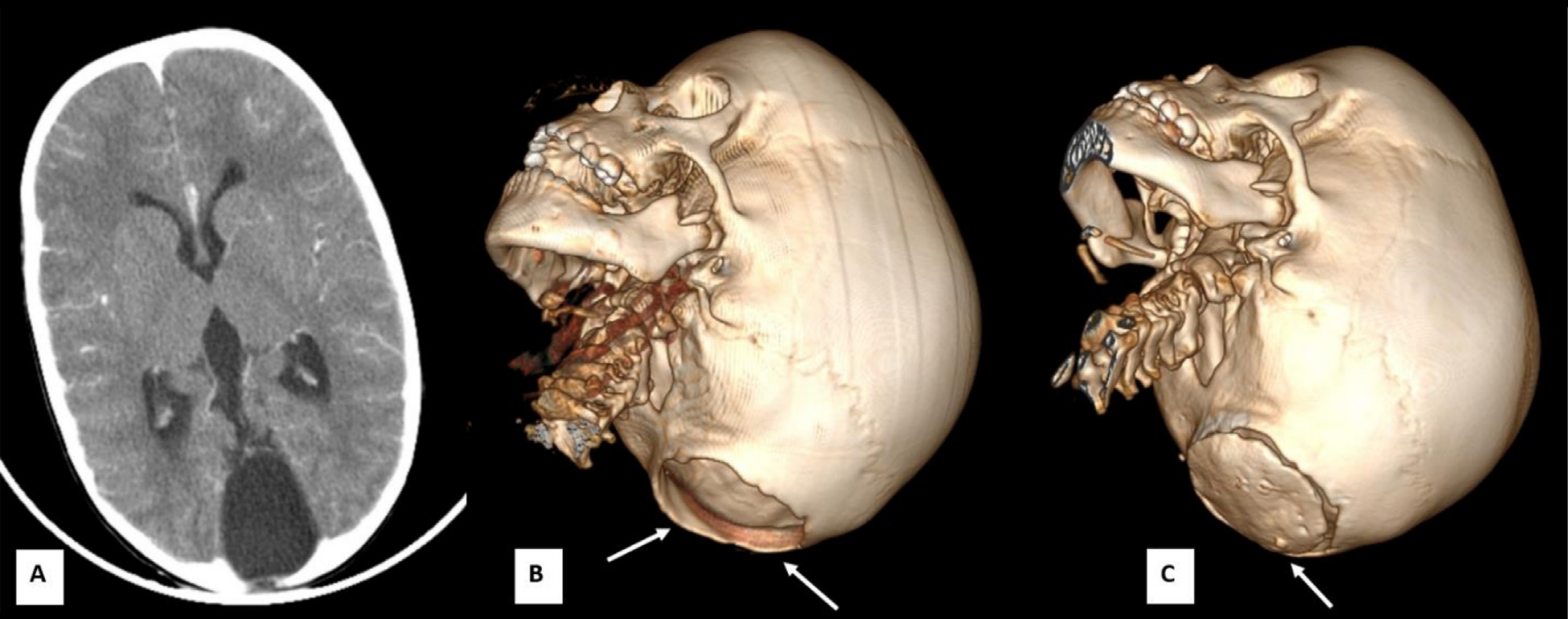
Joubert plus syndrome with posterior fossa cyst and occipital bone defect. (A) Axial contrast-enhanced CT image demonstrates a CSF-filled cystic structure in the posterior fossa, causing smooth remodeling and thinning of the surrounding occipital bone with focal dehiscence. (B) Preoperative 3D volumetric CT reconstruction shows a 6 × 3 cm bone defect in the midline occipital region (solid white arrows). (C) Postoperative 3D volumetric CT image reveals placement of a cranioplasty bone flap over the defect site (solid white arrow).

CT of the head was subsequently obtained to assess the integrity of the skull. It revealed a 6 × 3 cm occipital bone defect with herniation of a CSF-filled cystic structure, consistent with an occipital cephalocele (Fig. 3A and B).

Although there were no clinical signs of raised intracranial pressure, the presence of a herniated posterior fossa cyst through a sizeable occipital bone defect raised concern for potential complications. After neurosurgical consultation, the patient underwent occipital cranioplasty to repair the bone defect (Fig. 3C). The surgery was uneventful, and the patient recovered well postoperatively.

Following surgery, the patient was referred to the pediatric neurology clinic for regular follow-up and ongoing assessment of her neurological status. The family received a detailed explanation of the clinical findings, the nature of the disorder, and the importance of consistent follow-up care.

Due to the lack of access to genetic testing, the precise molecular classification of the disorder could not be determined. This was discussed with the family and documented as a limitation in the diagnostic workup.

## Discussion

Joubert syndrome is identified by a distinctive malformation at the junction of the midbrain and hindbrain, most notably producing a characteristic imaging feature known as the molar tooth sign, which is present in all individuals with the condition [5]. This radiologic sign is defined by deepening of the interpeduncular fossa, thickened and horizontally oriented superior cerebellar peduncles, and hypoplasia of the cerebellar vermis [6]. The absence of normal fiber crossing leads to both the enlargement of the peduncles and the increased depth of the interpeduncular cistern compared to a typical brain [7]. Vermian hypogenesis results in a prominent midline cleft that separates the normally developed cerebellar hemispheres [7]. In the present case, these features were evident on MRI, confirming the diagnosis of Joubert syndrome.

Dandy-Walker malformation is a rare congenital anomaly of the posterior fossa thought to result from abnormal development of the posterior membranous area during embryogenesis [8]. It has specific imaging findings, such as inferior-predominant vermian hypoplasia, vermian under-rotation, an obtuse fastigial recess, and inferolateral displacement of the choroid plexus and tela choroidea [8]. Other nonspecific imaging features, such as enlargement of the posterior fossa and upward displacement of the tentorium, can also be found [8].

Joubert syndrome typically presents with hypotonia, irregular eye movements, and respiratory disturbances. It may also involve multiple organ systems, including the kidneys, eyes, liver, and extremities [4]. Neurologically, it can be associated with other brain malformations such as hydrocephalus, abnormalities of the corpus callosum, pituitary agenesis, and, less commonly, cortical migration defects and occipital encephaloceles [4]. The latter finding was also observed in this case.

In our patient, the diagnosis of Dandy-Walker malformation was supported by the presence of a markedly elevated tegmentovermian angle, near complete absence of the inferior vermis, and a large posterior fossa cyst communicating with the fourth ventricle. These findings were accompanied by a midline cleft separating the interdigitated cerebellar hemispheres and the classic molar tooth sign formed by elongated and horizontally oriented superior cerebellar peduncles. These features indicate a rare combination of Joubert syndrome and Dandy-Walker malformation, a Joubert-Plus syndrome [1]. In addition, a calvarial defect with herniation of cerebrospinal fluid through the occipital bone was identified, which is consistent with cephalocele.

The coexistence of occipital encephalocele with Dandy-Walker malformation is a rare but documented association in the medical literature [9]. In contrast, the occurrence of occipital encephalocele alongside Joubert syndrome is exceedingly rare, with limited documentation in the current literature [3].

When evaluating posterior fossa anomalies with vermian hypoplasia and a cystic posterior fossa structure, it is essential to consider differential diagnoses. Rhombencephalosynapsis may mimic vermian agenesis but is characterized by fused cerebellar hemispheres without a cleft, unlike the interdigitating pattern seen in our patient [10]. Primary cerebellar hypoplasia typically presents with a uniformly small cerebellum without cystic enlargement of the fourth ventricle or associated midbrain anomalies [11]. Aarachnoid cysts and mega cisterna magna are cystic lesions that can resemble Dandy-Walker malformation but do not communicate with the fourth ventricle and usually have preserved vermian anatomy. Tectocerebellar dysplasia with encephalocele was excluded due to the absence of tectal fusion and significant brainstem hypoplasia beyond the typical features seen in Joubert syndrome [12]. Overall, the combination of imaging findings, including the molar tooth sign, cerebellar cleft, posterior fossa cyst communicating with the fourth ventricle, occipital bone defect, and absence of hydrocephalus, supports a diagnosis of Joubert Plus Syndrome with coexisting Dandy-Walker malformation and occipital cephalocele.

The management of Joubert-Plus syndrome is primarily supportive and tailored to the patient’s specific neurological and developmental needs, highlighting the importance of a coordinated, multidisciplinary approach and long-term follow-up to optimize outcomes [13].

## Conclusion

This case underlines the diagnostic utility of MRI and CT scans in differentiating overlapping congenital posterior fossa anomalies. Recognition of coexisting malformations is essential for prognosis and counseling.

## Patient consent

Informed consent was obtained from the patient’s family for publication of this case report.

## Author contributions

**T.M.** conducted the literature review and drafted the manuscript. **B.T.** collected and organized the clinical data. **R.G.** revised and edited the manuscript for intellectual content. All authors have read and approved the final version of the manuscript.

## Ethical approval

Ethical approval is not required for case reports at our institution.
